# Supplementary material for: Human detection of political speech deepfakes across transcripts, audio, and video
Source: Nat Commun. 2024 Sep 2;15:7629. doi: 10.1038/s41467-024-51998-z (PMC11368926; doi:10.1038/s41467-024-51998-z)
Supplement: Supplementary file 1 — Supplementary Information [file 41467_2024_51998_MOESM1_ESM.pdf]

Supplementary Information

	Dependent variable: Confidence Score		
	All	Real	Fabricated
Constant	57.66***	63.48***	51.40***
	(0.83)	(1.08)	(1.25)
Silent Video	6.56***	3.15*	10.45***
	(1.20)	(1.53)	(1.83)
Silent Video with Subtitles	8.73***	2.49	15.41***
	(1.12)	(1.52)	(1.69)
Audio	19.44***	11.35***	27.99***
	(1.17)	(1.48)	(1.68)
Audio with Subtitles	19.45***	10.50***	28.72***
	(1.05)	(1.36)	(1.58)
Video with Audio	25.23***	17.63***	33.30***
	(1.10)	(1.42)	(1.61)
Video with Audio and Subtitles	24.77***	14.95***	34.78***
	(1.08)	(1.49)	(1.53)
Number of Individuals	501	501	501
Observations	16,011	8,004	8,007
R^2	0.07	0.04	0.12
Note:	*p<0.05; **p<0.01; ***p<0.001		

Supplementary Table 1. Main Analysis for Experiment 1 Pre-registered main analysis for experiment 1a displaying ordinary least squares regressions with robust standard errors clustered on participant. Confidence score is the dependent variable, which is accuracy weighted by participants' confidence defined as the participant's confidence (ranging from 50 to 100) if correct and 100 minus the participant's confidence if incorrect. In each column, the independent variables indicate the modality with transcripts as the holdout. The first column shows all videos, the second column shows only real videos, and the third column shows only fabricated videos.

	Dependent variable: Accuracy		
	(1)	(2)	(3)
Constant	0.852***	0.832***	0.833***
	(0.044)	(0.050)	(0.020)
Voice Actor Videos (PDD)	-0.019		
	(0.047)		
Text-to-Speech Videos (PDD)	-0.130*		-0.111***
	(0.052)		(0.033)
Real Videos (PDD)	0.012	0.032	
	(0.050)	(0.055)	
Real Videos (Barari et al)	-0.020		
	(0.066)		
Number of Participants	302	302	302
Observations	5,964	2,383	2,390
R^2	0.018	0.002	0.018
Note:	*p<0.05; **p<0.01; ***p<0.001		

Supplementary Table 2. Main Analysis for Experiment 2 Pre-registered main analysis for Experiment 2 displaying ordinary least squares regressions with robust standard errors clustered on participants and stimuli. Accuracy is the dependent variable, which is a binary variable defined as 1 if participants accurately identify the stimuli as real and fake and 0 otherwise. In each column, the independent variables indicate the condition from which videos are drawn. In the first columns, the holdout condition is the fake videos used in Barari et al 2021. In the second column, the holdout condition is real videos used in Barari et al 2021. In the third column, the holdout condition is fake voice actor videos from the PDD.

Filename	Accuracy (TTS)	Accuracy (Voice Actor)	P-value	Significance (B-H)	Obs (TTS)	Obs (Voice Actor)
t-02	0.61	0.85	0.002	True	83	65
t-03	0.70	0.90	0.002	True	76	73
t-04	0.62	0.84	0.002	True	76	79
t-05	0.78	0.93	0.004	True	73	90
t-06	0.56	0.77	0.007	True	85	73
b-01	0.69	0.84	0.028	False	84	70
b-04	0.70	0.85	0.032	False	61	87
b-07	0.89	0.76	0.037	False	80	75
t-00	0.77	0.89	0.080	False	71	70
b-06	0.62	0.76	0.084	False	72	67
b-05	0.60	0.71	0.184	False	60	63
t-01	0.95	0.90	0.251	False	65	83
b-02	0.70	0.78	0.262	False	74	74
t-07	0.83	0.88	0.344	False	77	78
b-03	0.65	0.72	0.382	False	75	75
b-00	0.88	0.88	0.901	False	80	76

Supplementary Table 3. Text-to-Speech and Voice Actor Comparisons in Experiment 2 Pre-registered analysis comparing text-to-speech deepfakes videos to voice actor deepfake videos with p-values from two-sided t-tests and statistical significance based on controlling the false discovery rate using the Benjamini-Hochberg procedure. The last two columns indicate the number of observations for each text-to-speech deepfake and each voice actor deepfake.

	Correct Confidence	Response Time	Plays/Pauses
	(1)	(2)	(3)
Constant	0.650***	8.238***	0.125***
	(0.086)	(0.843)	(0.017)
Voice Actor Videos (PDD)	-0.047	-0.768	-0.004
	(0.090)	(0.854)	(0.017)
Text-to-Speech Videos (PDD)	-0.273**	0.970	0.023
	(0.099)	(0.870)	(0.017)
Real Videos (PDD)	-0.037	-0.113	-0.012
	(0.096)	(0.873)	(0.017)
Real Videos (Barari et al)	-0.106	1.915	0.042
	(0.120)	(0.981)	(0.034)
Number of Participants	302	302	302
Observations	5,964	5,964	5,964
R^2	0.024	0.009	0.003
Note:		*p<0.05; **p<0.01; ***p<0.001	

Supplementary Table 4. Secondary Analysis for Experiment 2 Pre-registered secondary analysis for Experiment 2 displaying ordinary least squares regressions with robust standard errors clustered on participants and stimuli. In column 1, "correct confidence" is the dependent variable, which is a defined as 1 - (5-confidence)/5 if participants accurately identify the stimuli as real and fake and -(confidence)/5 otherwise. In column 2, windsorized marginal response time is defined as the response time minus the duration of the stimulus windsorized at the 5% and 95% values. In column 3, the dependent variable is a binary variable for playing or pausing the video more than once. In each column, the independent variables indicate the condition from which videos are drawn and the holdout condition is the fake videos used in Barari et al 2021.

	Dependent variable: Accuracy					
	All	Real	Fabricated	All	Real	Fabricated
	(1)	(2)	(3)	(4)	(5)	(6)
Constant	0.587***	0.614***	0.577***	0.602***	0.617***	0.542***
	(0.033)	(0.041)	(0.036)	(0.033)	(0.040)	(0.049)
Audio	0.074***	0.034	0.112***	0.063**	0.041	0.152**
	(0.018)	(0.024)	(0.025)	(0.021)	(0.023)	(0.048)
Silent Video with Subtitles	0.042*	0.000	0.081**	0.010	-0.016	0.114**
	(0.018)	(0.013)	(0.031)	(0.017)	(0.016)	(0.042)
Video with Audio	0.165***	0.119***	0.209***	0.148***	0.117***	0.272***
	(0.021)	(0.025)	(0.030)	(0.024)	(0.026)	(0.049)
High Base Rate	-0.018	0.072***	-0.058***	-0.046	0.058*	-0.016
	(0.030)	(0.016)	(0.016)	(0.033)	(0.023)	(0.029)
Audio with Audio * High Base Rate				0.021	-0.033	-0.049
				(0.023)	(0.035)	(0.043)
Silent Video * High Base Rate				0.062*	0.075*	-0.042
				(0.027)	(0.034)	(0.035)
Video with Audio * High Base Rate				0.032	0.009	-0.078
				(0.028)	(0.028)	(0.045)
Number of Participants	1008	1008	1008	1008	1008	1008
Observations	19,812	9,707	10,105	19,812	9,707	10,105
R^2	0.017	0.014	0.026	0.017	0.016	0.027
Note:				*p<0.05; **p<0.01; ***p<0.001		

Supplementary Table 5. Main Analysis for Experiment 3 Pre-registered main analysis for experiment 3 displaying ordinary least squares regressions with robust standard errors clustered on participants and stimuli. Accuracy is the dependent variable, which is a binary variable defined as 1 if participants accurately identify the stimuli as real and fake and 0 otherwise. In each column, the independent variables indicate the stimuli's modality, assignment to the high-base rate of fakes condition, and an interaction between modality and high base rate. The held out conditions are transcripts and assignment to low base rate of fakes.

Filename	Accuracy (Transcript)	Accuracy (Silent Video)	P-value	Significance (B-H)	Obs (Transcript)	Obs (Silent Video)
t-01	0.55	0.80	0.000	True	187	155
b-01	0.41	0.59	0.001	True	143	150
t-00	0.65	0.82	0.001	True	159	153
b-06	0.41	0.59	0.001	True	142	147
t-06	0.32	0.50	0.002	True	141	149
b-00	0.80	0.66	0.003	True	144	148
t-02	0.36	0.51	0.009	True	163	152
b-05	0.58	0.71	0.014	False	157	163
b-05-p	0.85	0.73	0.016	False	144	160
t-05	0.41	0.54	0.018	False	148	156
b-04	0.45	0.59	0.018	False	150	154
b-02-p	0.32	0.43	0.061	False	143	153
b-07	0.53	0.63	0.068	False	182	157
b-00-p	0.55	0.63	0.160	False	153	136
t-07-p	0.75	0.69	0.189	False	156	171
t-07	0.74	0.68	0.281	False	130	154
t-04-p	0.46	0.52	0.313	False	162	157
b-03	0.69	0.64	0.362	False	148	163
t-03-p	0.70	0.66	0.513	False	161	162
b-04-p	0.74	0.72	0.621	False	171	131
b-02	0.46	0.49	0.623	False	149	146
t-00-p	0.54	0.56	0.674	False	142	163
t-02-p	0.70	0.72	0.705	False	147	165
b-06-p	0.78	0.79	0.737	False	151	153
t-05-p	0.64	0.66	0.753	False	165	139
b-03-p	0.55	0.57	0.768	False	159	149
b-01-p	0.54	0.55	0.788	False	165	160
t-06-p	0.68	0.69	0.790	False	174	156
b-07-p	0.35	0.33	0.791	False	150	149
t-01-p	0.84	0.85	0.795	False	155	162
t-04	0.61	0.60	0.874	False	168	135
t-03	0.41	0.42	0.954	False	163	171

Supplementary Table 6. Transcripts and Silent Videos Comparisons in Experiment 3 Pre-registered analysis for experiment 3 comparing transcripts to silent videos with p-values from two-sided t-tests and statistical significance based on controlling the false discovery rate using the Benjamini-Hochberg procedure. The last two columns indicate the number of observations.

Filename	Accuracy (Transcript)	Accuracy (Audio Only)	P-value	Significance (B-H)	Obs (Transcript)	Obs (Audio Only)
t-01	0.55	0.74	0.000	True	187	151
t-06-p	0.68	0.85	0.000	True	143	129
b-02-p	0.32	0.56	0.000	True	159	151
t-05	0.41	0.66	0.000	True	142	165
t-03	0.41	0.72	0.000	True	141	164
t-02	0.36	0.60	0.000	True	144	143
b-01	0.41	0.59	0.001	True	163	165
t-05-p	0.64	0.79	0.004	True	157	151
b-07	0.53	0.68	0.006	True	144	149
b-04-p	0.74	0.60	0.007	True	148	159
t-01-p	0.84	0.92	0.022	False	150	150
t-07	0.74	0.84	0.029	False	143	164
b-04	0.45	0.57	0.042	False	182	144
t-02-p	0.70	0.59	0.049	False	153	154
b-06-p	0.78	0.86	0.072	False	156	139
t-06	0.32	0.41	0.092	False	130	147
b-00	0.80	0.86	0.155	False	162	139
b-07-p	0.35	0.43	0.162	False	148	143
t-04	0.61	0.67	0.225	False	161	170
t-00-p	0.54	0.61	0.243	False	171	161
b-06	0.41	0.47	0.338	False	149	174
b-05-p	0.85	0.82	0.591	False	142	145
t-00	0.65	0.68	0.609	False	147	161
b-03-p	0.55	0.58	0.613	False	151	163
b-02	0.46	0.49	0.641	False	165	161
t-03-p	0.70	0.67	0.697	False	159	162
b-05	0.58	0.56	0.728	False	165	161
b-03	0.69	0.71	0.755	False	174	135
t-07-p	0.75	0.76	0.864	False	150	160
b-00-p	0.55	0.54	0.872	False	155	148
b-01-p	0.54	0.53	0.890	False	168	165
t-04-p	0.46	0.46	0.945	False	163	156

Supplementary Table 7. Transcripts and Audios Comparisons in Experiment 3 Pre-registered analysis for experiment 3 comparing transcripts to audio with p-values from two-sided t-tests and statistical significance based on controlling the false discovery rate using the Benjamini-Hochberg procedure. The last two columns indicate the number of observations.

Filename	Accuracy (Transcript)	Accuracy (Video with Audio)	P-value	Significance (B-H)	Obs (Transcript)	Obs (Video with Audio)
t-05-p	0.64	0.87	0.000	True	187	151
t-00-p	0.54	0.80	0.000	True	143	168
b-07	0.53	0.83	0.000	True	159	158
t-03	0.41	0.65	0.000	True	142	154
t-05	0.41	0.81	0.000	True	141	166
t-01	0.55	0.84	0.000	True	144	167
b-04	0.45	0.75	0.000	True	163	156
t-02	0.36	0.68	0.000	True	157	146
t-06-p	0.68	0.86	0.000	True	144	177
b-02-p	0.32	0.62	0.000	True	148	144
b-02	0.46	0.71	0.000	True	150	169
b-01	0.41	0.78	0.000	True	143	132
b-00-p	0.55	0.80	0.000	True	182	149
b-05	0.58	0.76	0.001	True	153	159
b-01-p	0.54	0.72	0.001	True	156	160
t-07	0.74	0.89	0.001	True	130	146
t-00	0.65	0.81	0.001	True	162	166
t-06	0.32	0.49	0.001	True	148	148
b-07-p	0.35	0.52	0.003	True	161	134
b-06	0.41	0.56	0.009	True	171	145
b-00	0.80	0.90	0.012	True	149	175
b-04-p	0.74	0.84	0.042	False	142	161
t-01-p	0.84	0.91	0.051	False	147	176
t-04-p	0.46	0.57	0.057	False	151	151
b-06-p	0.78	0.86	0.077	False	165	168
t-07-p	0.75	0.83	0.101	False	159	146
t-04	0.61	0.69	0.107	False	165	157
t-03-p	0.70	0.62	0.183	False	174	140
b-03-p	0.55	0.60	0.394	False	150	174
t-02-p	0.70	0.73	0.666	False	155	160
b-05-p	0.85	0.86	0.819	False	168	152
b-03	0.69	0.70	0.916	False	163	137

Supplementary Table 8. Transcripts and Videos Comparisons in Experiment 3 Pre-registered analysis for experiment 3 comparing transcripts to video with audio with p-values from two-sided t-tests and statistical significance based on controlling the false discovery rate using the Benjamini-Hochberg procedure. The last two columns indicate the number of observations.

Filename	Accuracy (Silent Video)	Accuracy (Video with Audio)	P-value	Significance (B-H)	Obs (Silent Video)	Obs (Video with Audio)
b-00	0.66	0.90	0.000	True	155	151
t-06-p	0.69	0.86	0.000	True	150	168
b-01	0.59	0.78	0.000	True	153	158
t-05-p	0.66	0.87	0.000	True	147	154
b-02	0.49	0.71	0.000	True	149	166
t-05	0.54	0.81	0.000	True	148	167
t-03	0.42	0.65	0.000	True	152	156
t-00-p	0.56	0.80	0.000	True	163	146
t-07	0.68	0.89	0.000	True	160	177
b-07	0.63	0.83	0.000	True	156	144
b-07-p	0.33	0.52	0.001	True	154	169
b-00-p	0.63	0.80	0.001	True	153	132
b-02-p	0.43	0.62	0.001	True	157	149
b-04	0.59	0.75	0.001	True	136	159
b-01-p	0.55	0.72	0.002	True	171	160
t-02	0.51	0.68	0.002	True	154	146
t-07-p	0.69	0.83	0.004	True	157	166
b-05-p	0.73	0.86	0.010	True	163	148
b-04-p	0.72	0.84	0.011	True	162	134
t-04	0.60	0.69	0.089	False	131	145
t-01-p	0.85	0.91	0.108	False	146	175
b-06-p	0.79	0.86	0.167	False	163	161
t-01	0.80	0.84	0.299	False	165	176
b-03	0.64	0.70	0.315	False	153	151
t-04-p	0.52	0.57	0.374	False	139	168
b-05	0.71	0.76	0.381	False	149	146
t-03-p	0.66	0.62	0.496	False	160	157
b-06	0.59	0.56	0.534	False	156	140
b-03-p	0.57	0.60	0.568	False	149	174
t-02-p	0.72	0.73	0.955	False	162	160
t-00	0.82	0.81	0.963	False	135	152
t-06	0.50	0.49	0.966	False	171	137

Supplementary Table 9. Silent Videos and Video Comparisons in Experiment 3 Pre-registered analysis for experiment 3 comparing silent video to video with audio with p-values from two-sided t-tests and statistical significance based on controlling the false discovery rate using the Benjamini-Hochberg procedure. The last two columns indicate the number of observations.

Filename	Accuracy (Silent Video)	Accuracy (Audio Only)	P-value	Significance (B-H)	Obs (Silent Video)	Obs (Audio Only)
b-00	0.66	0.86	0.000	True	155	151
t-03	0.42	0.72	0.000	True	150	129
t-06-p	0.69	0.85	0.001	True	153	151
t-07	0.68	0.84	0.002	True	147	165
b-05	0.71	0.56	0.005	True	149	164
t-00	0.82	0.68	0.006	True	148	143
t-05-p	0.66	0.79	0.012	False	152	165
t-02-p	0.72	0.59	0.015	False	163	151
t-05	0.54	0.66	0.020	False	160	149
b-02-p	0.43	0.56	0.022	False	156	159
b-04-p	0.72	0.60	0.024	False	154	150
b-06	0.59	0.47	0.027	False	153	164
b-05-p	0.73	0.82	0.051	False	157	144
t-01-p	0.85	0.92	0.054	False	136	154
b-07-p	0.33	0.43	0.082	False	171	139
t-02	0.51	0.60	0.108	False	154	147
b-00-p	0.63	0.54	0.125	False	157	139
t-06	0.50	0.41	0.139	False	163	143
t-07-p	0.69	0.76	0.142	False	162	170
b-06-p	0.79	0.86	0.157	False	131	161
t-04	0.60	0.67	0.187	False	146	174
t-01	0.80	0.74	0.191	False	163	145
b-03	0.64	0.71	0.222	False	165	161
b-07	0.63	0.68	0.290	False	153	163
t-04-p	0.52	0.46	0.344	False	139	161
t-00-p	0.56	0.61	0.438	False	149	162
b-01-p	0.55	0.53	0.676	False	160	161
b-04	0.59	0.57	0.763	False	156	135
t-03-p	0.66	0.67	0.782	False	149	160
b-03-p	0.57	0.58	0.827	False	162	148
b-01	0.59	0.59	0.924	False	135	165
b-02	0.49	0.49	0.970	False	171	156

Supplementary Table 10. Silent Videos and Audios Comparisons in Experiment 3 Pre-registered analysis for experiment 3 comparing silent video to audio with p-values from two-sided t-tests and statistical significance based on controlling the false discovery rate using the Benjamini-Hochberg procedure. The last two columns indicate the number of observations.

Filename	Accuracy (Audio Only)	Accuracy (Video with Audio)	P-value	Significance (B-H)	Obs (Audio Only)	Obs (Video with Audio)
b-04-p	0.60	0.84	0.000	True	151	151
b-00-p	0.54	0.80	0.000	True	129	168
b-01	0.59	0.78	0.000	True	151	158
b-01-p	0.53	0.72	0.000	True	165	154
b-02	0.49	0.71	0.000	True	164	166
t-00-p	0.61	0.80	0.000	True	143	167
b-05	0.56	0.76	0.000	True	165	156
b-04	0.57	0.75	0.001	True	151	146
b-07	0.68	0.83	0.003	True	149	177
t-05	0.66	0.81	0.003	True	159	144
t-00	0.68	0.81	0.006	True	150	169
t-02-p	0.59	0.73	0.013	True	164	132
t-01	0.74	0.84	0.023	False	144	149
t-04-p	0.46	0.57	0.065	False	154	159
t-05-p	0.79	0.87	0.080	False	139	160
b-07-p	0.43	0.52	0.116	False	147	146
b-06	0.47	0.56	0.117	False	139	166
t-06	0.41	0.49	0.135	False	143	148
t-02	0.60	0.68	0.137	False	170	134
t-07-p	0.76	0.83	0.143	False	161	145
t-07	0.84	0.89	0.184	False	174	175
t-03	0.72	0.65	0.186	False	145	161
b-00	0.86	0.90	0.288	False	161	176
b-02-p	0.56	0.62	0.308	False	163	151
t-03-p	0.67	0.62	0.333	False	161	168
b-05-p	0.82	0.86	0.447	False	162	146
t-04	0.67	0.69	0.703	False	161	157
b-03-p	0.58	0.60	0.727	False	135	140
t-01-p	0.92	0.91	0.781	False	160	174
b-03	0.71	0.70	0.839	False	148	160
t-06-p	0.85	0.86	0.904	False	165	152
b-06-p	0.86	0.86	0.964	False	156	137

Supplementary Table 11. Audios and Videos Comparisons in Experiment 3 Pre-registered analysis for experiment 3 comparing audio to video with audio with p-values from two-sided t-tests and statistical significance based on controlling the false discovery rate using the Benjamini-Hochberg procedure. The last two columns indicate the number of observations.

	Correct Confidence	Response Time	Plays/Pauses
	(1)	(2)	(3)
Constant	0.152**	10.813***	0.098***
	(0.050)	(0.764)	(0.008)
Audio	0.115***		
	(0.031)		
Silent Video	0.019	6.744***	0.151***
	(0.027)	(1.839)	(0.012)
Video with Audio	0.254***	1.909	0.030***
	(0.036)	(1.122)	(0.009)
High Base Rate	-0.061	-0.817	-0.004
	(0.049)	(0.933)	(0.011)
Audio with Audio * High Base Rate	0.026		
	(0.035)		
Silent Video * High Base Rate	0.093*	-1.259	0.009
	(0.042)	(2.057)	(0.015)
Video with Audio * High Base Rate	0.058	0.818	0.019
	(0.045)	(1.354)	(0.014)
Number of Participants	1008	1008	1008
Observations	19,812	14,838	14,838
R^2	0.023	0.003	0.032

Note: *p<0.05; **p<0.01; ***p<0.001

Supplementary Table 12. Secondary Analysis in Experiment 3 Pre-registered secondary analysis for experiment 3 displaying ordinary least squares regressions with robust standard errors clustered on participants and stimuli. In column 1, "correct confidence" is the dependent variable, which is a defined as 1 - (5-confidence)/5 if participants accurately identify the stimuli as real and fake and -(confidence)/5 otherwise. In column 2, windsorized marginal response time is defined as the response time minus the duration of the stimulus windsorized at the 5% and 95% values. In column 3, the dependent variable is a binary variable for playing or pausing the video more than once. In each column, the independent variables indicate the stimuli's modality, assignment to the high-base rate of fakes condition, and an interaction between modality and high base rate. The held out conditions are transcripts in columns 1 and audio in columns 2 and 3 and assignment to low base rate of fakes in all columns.

	Dependent variable: Accuracy
	Correct
	(1)
Constant	0.756***
	(0.031)
Voice Actor	0.092**
	(0.034)
Video with Audio	0.088**
	(0.030)
Voice Actor * Video with Audio	-0.187***
	(0.044)
Number of Participants	206
Observations	3,215
R^2	0.014
Note:	*p<0.05; **p<0.01; ***p<0.001

Supplementary Table 13. Main Analysis in Experiment 4 Pre-registered main analysis for experiment 4 displaying ordinary least squares regressions with robust standard errors clustered on participants and stimuli. Accuracy is the dependent variable, which is a binary variable defined as 1 if participants accurately identify the stimuli as real and fake and 0 otherwise. In each column, the independent variables indicate whether the stimulus was from the voice actor, whether the stimulus was shown as an audio or video with audio, and an interaction between these two variables.

Filename	Accuracy (Voice Actor)	Accuracy (Real)	P-value	Significance (B-H)	Obs (Voice Actor)	Obs (Real)
b-05-p	0.51	0.91	0.000	True	45	62
b-06-p	0.66	0.95	0.000	True	54	56
t-03-p	0.93	0.55	0.000	True	54	53
t-01-p	0.71	0.96	0.001	True	54	55
t-06-p	0.72	0.94	0.001	True	65	47
b-00-p	0.60	0.85	0.002	True	57	54
t-07-p	0.69	0.92	0.002	True	47	60
b-04-p	0.78	0.98	0.003	True	57	53
t-05-p	0.72	0.89	0.026	True	57	52
b-02-p	0.72	0.87	0.063	False	62	47
t-04-p	0.82	0.69	0.105	False	60	49
b-07-p	0.89	0.79	0.141	False	67	42
b-03-p	0.80	0.67	0.147	False	56	51
t-02-p	0.68	0.80	0.189	False	47	63
t-00-p	0.86	0.85	0.844	False	43	66
b-01-p	0.80	0.80	0.925	False	48	61

Supplementary Table 14. Voice Actor and Original Speaker in Videos Comparisons in Experiment 4 Pre-registered analysis comparing real videos with voice actor audio to real videos with the original speaker's audio with p-values from two-sided t-tests and statistical significance based on controlling the false discovery rate using the Benjamini-Hochberg procedure. The last two columns indicate the number of observations for each text-to-speech deepfake and each voice actor deepfake.

Filename	Accuracy (Voice Actor)	Accuracy (Real)	P-value	Significance (B-H)	Obs (Voice Actor)	Obs (Real)
b-02-p	0.93	0.51	0.000	True	49	44
t-00-p	0.93	0.64	0.001	True	46	47
b-07-p	0.87	0.70	0.038	False	54	37
t-04-p	0.88	0.70	0.040	False	51	41
b-00-p	0.82	0.64	0.051	False	47	44
t-02-p	0.78	0.59	0.056	False	43	48
b-03-p	0.82	0.68	0.119	False	48	45
t-07-p	0.85	0.93	0.221	False	47	46
t-05-p	0.86	0.79	0.339	False	46	45
t-03-p	0.76	0.72	0.639	False	47	43
b-06-p	0.83	0.87	0.657	False	49	44
t-06-p	0.90	0.92	0.728	False	34	57
b-05-p	0.81	0.83	0.811	False	42	47
b-01-p	0.83	0.81	0.829	False	37	56
b-04-p	0.85	0.86	0.866	False	41	52
t-01-p	0.83	0.84	0.926	False	52	42

Supplementary Table 15. Voice Actor and Original Speaker in Audios Comparisons in Experiment 4 Pre-registered analysis comparing voice actor audio to the original speaker's audio with p-values from two-sided t-tests and statistical significance based on controlling the false discovery rate using the Benjamini-Hochberg procedure. The last two columns indicate the number of observations for each text-to-speech deepfake and each voice actor deepfake.

	Correct Confidence	Response Time	Plays/Pauses
	(1)	(2)	(3)
Constant	0.425^{***}	7.847^{***}	0.068^{***}
	(0.053)	(0.509)	(0.015)
Voice Actor	0.186^{**}	-1.539^{***}	-0.001
	(0.059)	(0.408)	(0.015)
Video with Audio	0.154^{**}	-0.658	0.007
	(0.051)	(0.695)	(0.020)
Voice Actor * Video with Audio	-0.325^{***}	0.998	0.020
	(0.075)	(0.600)	(0.022)
Number of Participants	206	206	206
Observations	3,215	3,215	3,215
R^2	0.016	0.005	0.002
Note:			$^{*}p<0.05$; $^{**}p<0.01$; $^{***}p<0.001$

Supplementary Table 16. Secondary Analysis in Experiment 4 Pre-registered secondary analysis for experiment 4 displaying ordinary least squares regressions with robust standard errors clustered on participants and stimuli. In column 1, "correct confidence" is the dependent variable, which is a defined as 1 - (5-confidence)/5 if participants accurately identify the stimuli as real and fake and -(confidence)/5 otherwise. In column 2, windsorized marginal response time is defined as the response time minus the duration of the stimulus windsorized at the 5% and 95% values. In column 3, the dependent variable is a binary variable for playing or pausing the video more than once. In each column, the independent variables indicate whether the stimulus was from the voice actor, whether the stimulus was shown as an audio or video with audio, and an interaction between these two variables.

	Accurate Suspicion of Fake			Inaccurate Suspicion of Fake		
	(1)	(2)	(3)	(4)	(5)	(6)
Constant	0.073***	0.035	0.056*	0.048**	0.017	0.031
	(0.017)	(0.026)	(0.026)	(0.017)	(0.017)	(0.018)
Silent Video	0.102**	0.103**	0.040	0.012	0.014	-0.024
	(0.032)	(0.032)	(0.054)	(0.022)	(0.022)	(0.019)
Audio	0.058*	0.063*	0.072	0.038	0.037	0.032
	(0.028)	(0.027)	(0.038)	(0.029)	(0.029)	(0.029)
Video	0.252***	0.251***	0.213***	0.013	0.011	0.001
	(0.034)	(0.034)	(0.037)	(0.025)	(0.025)	(0.022)
Primed		0.074	0.032		0.063***	0.035
		(0.039)	(0.038)		(0.019)	(0.030)
Primed * Silent Video			0.127*			0.079
			(0.064)			(0.041)
Primed * Audio			-0.027			0.010
			(0.056)			(0.047)
Primed * Video			0.076			0.022
			(0.071)			(0.043)
Number of Participants	200	200	200	200	200	200
Observations	1,018	1,018	1,018	982	982	982
R^2	0.060	0.069	0.076	0.003	0.020	0.024
Note:				*p<0.05; **p<0.01; ***p<0.001		

Supplementary Table 17. Main Analysis for Experiment 5 Pre-registered main analysis for experiment 5 displaying ordinary least squares regressions with robust standard errors clustered on participants and stimuli. Accurate suspicion of a fake (true positive) is the dependent variable in columns 1 through 3 and inaccurate suspicion of a fake (false positive) is the dependent variable in columns 4 through 6. The independent variables indicate the stimuli's modality (Silent Video, Audio, and Video), assignment to seeing the deepfake attention check as the first stimulus (Primed), and interactions between these independent variables. The held out condition in this regression is the stimuli appearing as text transcripts.
