# Supplementary material for: Human detection of political speech deepfakes across transcripts, audio, and video
Source: Nat Commun. 2024 Sep 2;15:7629. doi: 10.1038/s41467-024-51998-z (PMC11368926; doi:10.1038/s41467-024-51998-z)
Supplement: Supplementary file 3 — Reporting Summary [file 41467_2024_51998_MOESM3_ESM.pdf]

nature portfolio

Corresponding author(s): Matthew Groh

Last updated by author(s): 8/19/2024

Reporting Summary

Nature Portfolio wishes to improve the reproducibility of the work that we publish. This form provides structure for consistency and transparency in reporting. For further information on Nature Portfolio policies, see our Editorial Policies and the Editorial Policy Checklist.

Statistics

For all statistical analyses, confirm that the following items are present in the figure legend, table legend, main text, or Methods section.

n/a	Confirmed	
☐	☒	The exact sample size (*n*) for each experimental group/condition, given as a discrete number and unit of measurement
☐	☒	A statement on whether measurements were taken from distinct samples or whether the same sample was measured repeatedly
☐	☒	The statistical test(s) used AND whether they are one- or two-sided<br>*Only common tests should be described solely by name; describe more complex techniques in the Methods section.*
☐	☒	A description of all covariates tested
☐	☒	A description of any assumptions or corrections, such as tests of normality and adjustment for multiple comparisons
☐	☒	A full description of the statistical parameters including central tendency (e.g. means) or other basic estimates (e.g. regression coefficient) AND variation (e.g. standard deviation) or associated estimates of uncertainty (e.g. confidence intervals)
☐	☒	For null hypothesis testing, the test statistic (e.g. *F*, *t*, *r*) with confidence intervals, effect sizes, degrees of freedom and *P* value noted<br>*Give P values as exact values whenever suitable.*
☒	☐	For Bayesian analysis, information on the choice of priors and Markov chain Monte Carlo settings
☒	☐	For hierarchical and complex designs, identification of the appropriate level for tests and full reporting of outcomes
☐	☒	Estimates of effect sizes (e.g. Cohen's *d*, Pearson's *r*), indicating how they were calculated

Our web collection on statistics for biologists contains articles on many of the points above.

Software and code

Policy information about availability of computer code

Data collection	All data is collected via the "Detect Fakes" website, which is served with NGINX and Flask, a micro web framework written in Python. All data is stored in a SQL database, and we copied the SQL database tables to .csvs.
Data analysis	Data analysis is done in Python 3.11 with the following libraries: pandas numpy scipy statsmodels stargazer. All code produced to analyze the data generated in this study are available on Zenodo at https://zenodo.org/doi/10.5281/zenodo.13340207.

For manuscripts utilizing custom algorithms or software that are central to the research but not yet described in published literature, software must be made available to editors and reviewers. We strongly encourage code deposition in a community repository (e.g. GitHub). See the Nature Portfolio guidelines for submitting code & software for further information.

Data

Policy information about availability of data

All manuscripts must include a data availability statement. This statement should provide the following information, where applicable:

- Accession codes, unique identifiers, or web links for publicly available datasets
- A description of any restrictions on data availability
- For clinical datasets or third party data, please ensure that the statement adheres to our policy

The participant response data generated in this study have been deposited in Zenodo at https://zenodo.org/doi/10.5281/zenodo.13340207. The stimuli data are

available under restricted access due to the sensitivity of the topic and potential misuse of stimulus materials, access can be obtained upon request to registered Zenodo users at https://zenodo.org/doi/10.5281/zenodo.12709664.

Research involving human participants, their data, or biological material

Policy information about studies with human participants or human data. See also policy information about sex, gender (identity/presentation), and sexual orientation and race, ethnicity and racism.

Reporting on sex and gender	In these experiments, we do not find consistent differences based on sex, and we do not report sex-based analyses because we did not pre-register sex-based analyses nor do we have theoretical grounds for suspecting differences across sex.
Reporting on race, ethnicity, or other socially relevant groupings	We do not do any analyses related to race or ethnicity, and we mention race only to demonstrate that our sample is generally representative of the United States demographics. Race is self-reported on the Prolific platform.
Population characteristics	See above
Recruitment	The research sample consisted of 2,215 participants recruited from Prolific and 41,313 non-recruited participant who found the experiment organically via search engines or media.. We include both samples of participants to demonstrate the results are robust to crowdworkers on either side of the political spectrum in America and people on the Internet who are interested in learning about misinformation. A potential self-selection biases may include people who are interested in participating in online research, but it is unclear how this self-selection may influence the results.
Ethics oversight	This research complied with all relevant ethical regulations and the Massachusetts Institute of Technology's Committee on the Use of Humans as Experimental Subjects approved this study as Exempt Category 3 – Benign Behavioral Intervention. This study's exemption identification numbers are E-3105, E-3354, E-4735, and E-5493.

Note that full information on the approval of the study protocol must also be provided in the manuscript.

Field-specific reporting

Please select the one below that is the best fit for your research. If you are not sure, read the appropriate sections before making your selection.

☐ Life sciences ☒ Behavioural & social sciences ☐ Ecological, evolutionary & environmental sciences

For a reference copy of the document with all sections, see nature.com/documents/nr-reporting-summary-flat.pdf

Behavioural & social sciences study design

All studies must disclose on these points even when the disclosure is negative.

Study description	This is a quantitative research study based on a randomized experiment conducted on a custom designed website for distinguishing between fabricated and non-fabricated media and Qualtrics for collecting participants comments on the stimuli.
Research sample	The research sample consisted of 2,215 participants recruited from Prolific and 41,313 non-recruited participant who found the experiment organically via search engines or media. All participants from Prolific are from the United States, and we recruited participants from Prolific because Prolific has a diverse pool of people interested in engaging in online research studies. The participant population from Prolific is balanced across sex and is close to representative of demographics in the United States across age and race. Participants from Prolific range in age from 18 to 78 with a median of 48. 76% of non-recruited participants visited from outside the United States. We include these samples of participants to demonstrate the results are robust to crowdworkers on either side of the political spectrum in the United States and also people on the Internet who are interested in learning about misinformation. In these experiments, we do not find consistent differences based on sex, and we do not report sex-based analyses because we did not pre-register sex-based analyses nor do we have theoretical grounds for suspecting differences across sex.
Sampling strategy	We conducted 5 pre-registered experiments to recruit 2,215 participants from Prolific. In experiment 1, the sample size of 500 participants providing responses to 32 stimuli each provides 16,000 observations split between 7 conditions, which provides over 90% statistical power to detect differences between conditions of 5 percentage points. All following experiments keep this statistical power in mind. The research sample from Prolific and non-recruited participants is based on convenience sampling.
Data collection	We collected data based on participants' interaction with the user interface. The participants engaged with the experiment remotely and no researcher was present with the participants.
Timing	We started collecting data on March 4, 2021 and ended on December 20, 2023
Data exclusions	We include analysis with and without data exclusions to demonstrate robustness. In experiment 1, our main analysis focuses on three pre-registered exclusions: (1) people who disagree to do the experiment in good faith, (2) people who fail the simple attention check at the beginning of the experiment, and (3) people who select the default "fakeness" setting (i.e just as likely a fabricated as not) on more than 10 media objects. These exclusion criteria resulted in 509 participants from Prolific. All following experiments follow this exclusion pattern.

Non-participation	In experiment 1, 606 participants visited the Detect Fakes experiment from Prolific, 38 responded they could not answer faithfully, 12 participants withdrew from the experiment, 2 participants did not continue past the initial survey, and 554 participated in the deepfake detection experiment. In experiment 2, 30 participants fail the attention check. 3n experiment 3, 59 participants fail the attention check. In experiment 4, 14 participants fail the attention check and 1 withdrew. In experiment 5, 3 participants fail the attention check and 21 participants withdrew before completing the experiment.
Randomization	We conducted randomization at the stimuli level. The order of the 32 stimuli was randomly assigned to each participant, and the modality of each stimuli was randomly assigned to one of seven conditions (text only, audio only, video only, text + audio, text + video, text + audio + video, audio + video) for each participant.

Reporting for specific materials, systems and methods

We require information from authors about some types of materials, experimental systems and methods used in many studies. Here, indicate whether each material, system or method listed is relevant to your study. If you are not sure if a list item applies to your research, read the appropriate section before selecting a response.

Materials & experimental systems

n/a	Involved in the study
☒	☐ Antibodies
☒	☐ Eukaryotic cell lines
☒	☐ Palaeontology and archaeology
☒	☐ Animals and other organisms
☒	☐ Clinical data
☒	☐ Dual use research of concern
☒	☐ Plants

Methods

n/a	Involved in the study
☒	☐ ChIP-seq
☒	☐ Flow cytometry
☒	☐ MRI-based neuroimaging

Plants

Seed stocks	N/A
Novel plant genotypes	N/A
Authentication	N/A
